# Longevity of System Functions in Biology and Biomimetics: A Matter of Robustness and Resilience

**DOI:** 10.3390/biomimetics8020173

**Published:** 2023-04-21

**Authors:** Max D. Mylo, Olga Speck

**Affiliations:** 1 Cluster of Excellence livMatS @ FIT—Freiburg Center for Interactive Materials and Bioinspired Technologies, University of Freiburg, Georges-Köhler-Allee 105, 79110 Freiburg, Germany; olga.speck@biologie.uni-freiburg.de; 2 Department of Microsystems Engineering—IMTEK, University of Freiburg, Georges-Köhler-Allee 103, 79110 Freiburg, Germany; 3 Plant Biomechanics Group @ Botanic Garden Freiburg, University of Freiburg, Schänzlestr. 1, 79104 Freiburg, Germany

**Keywords:** lifespan and lifetime, material system, responsive materials, redundancy, safety factor, self-repair, sustainable development goal (SDG), trained materials

## Abstract

Within the framework of a circular economy, we aim to efficiently use raw materials and reduce waste generation. In this context, the longevity of biomimetic material systems can significantly contribute by providing robustness and resilience of system functionality inspired by biological models. The aim of this review is to outline various principles that can lead to an increase in robustness (e.g., safety factor, gradients, reactions to environmental changes) and resilience (e.g., redundancy, self-repair) and to illustrate the principles with meaningful examples. The study focuses on plant material systems with a high potential for transfer to biomimetic applications and on existing biomimetic material systems. Our fundamental concept is based on the functionality of the entire system as a function of time. We use functionality as a dimensionless measure of robustness and resilience to quantify the system function, allowing comparison within biological material systems and biomimetic material systems, but also between them. Together with the enclosed glossary of key terms, the review provides a comprehensive toolbox for interdisciplinary teams. Thus, allowing teams to communicate unambiguously and to draw inspiration from plant models when developing biomimetic material systems with great longevity potential.

## 1. Introduction

### 1.1. Longevity—More than a Prominent Catchphrase

In the wake of increasing media attention given to climate change and consumer awareness and alongside the growing demand for a sustainable economy, increasing numbers of companies are using the catchphrase “longevity” to advertise their products and services. In this context, the longevity of novel consumer products is almost exclusively presented as being positive and desirable, although neither the way in which this is defined or measured, nor whether the promise is accompanied by positive effects is actually addressed. Moreover, the term is also widely used in the life sciences, particularly in the context of healthy human ageing and the resulting increase in life expectancy in recent decades (life expectancy has at least doubled in almost all countries since the mid-19th century [1]). However, the ageing effects and external environmental influences that inevitably lead to the mortality of any individual are of course not unique to humans, but affect all kind of living organisms.

**Figure 1:**
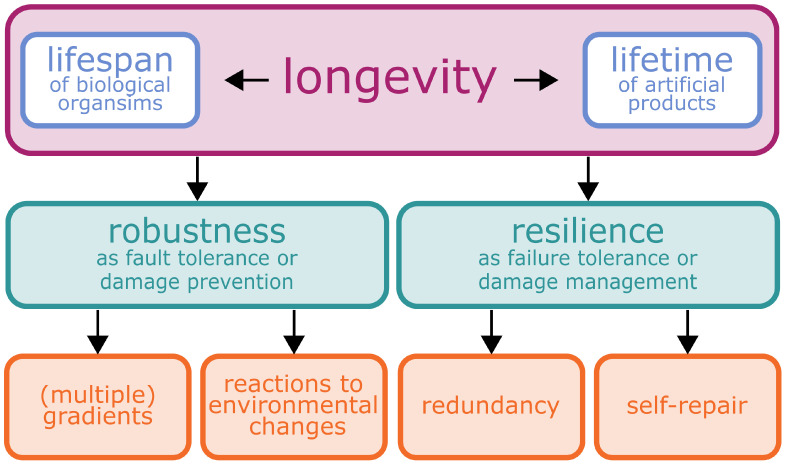
Overview of key terms. Longevity is measured as “lifespan” in biology and “lifetime” in technology. The concepts of robustness and resilience are based on various principles. The system functions can be quantified by the percentage functionality of the entire system.

Before presenting selected concepts that can lead to an enhancement of the longevity potential of material systems and functions, we will first discuss the ways in which the term “longevity” and its characteristics in the context of “lifespan” of plant material systems and “lifetime” of artificial material systems can be understood. An overview of the relationship between longevity, the concepts of “robustness” and “resilience”, and the respective principles is given in Figure 1.

The fundamental concept of our study is based on the functionality of entire material systems in various states after perturbations. We have chosen functionality as a measure of robustness (cf. Section 2) and resilience (cf. Section 3) because it can reflect the quality of a selected function as a dimensionless value or percentage. Thus, we are able to compare loss and regain of functionality in various states within biological and artificial material systems, but also make comparisons between them. The latter is particularly important in the context of transferring functional principles from plants and animals to biomimetic applications [2]. A prime example for resilience is the quantification of self-repair efficiency (cf. Section 3.2). This is done using various equations that calculate dimensionless quantities by comparing properties of various states (freshly damaged, sealed or healed) with respect to the intact undamaged state. Finally, a major advantage of our fundamental concept is that both robustness and resilience can be represented by means of system functionality as a function of time and can thus be both compared and combined with each other.

#### 1.1.1. Lifespan as a Longevity Measure in Plants

The longevity of each living being is limited by its death and is commonly quantified by its lifespan, which is determined to a large extent by its genome and is influenced by its environment. In addition to these similarities between plants, animals and humans, certain differences are obvious, such as the way in which they produce energy or reproduce. Based on our expertise, we will focus on plant material systems in this paper when discussing biological longevity.

During their lifetime, flowering plants go through a defined, genetically predetermined cycle that consists of germination, growth, flower development, pollination, the production of seeds (and in some species, vegetative reproduction) and senescence [3]. Although this cycle ensures reproduction and the survival of the species, the duration and number of occurrences of each step, except of course for germination and whole-plant senescence, can vary from species to species [4]. For example, in some plants, all these processes take place within only one year, in which case the plants are designated as annual (such as corn and tomatoes) or within two years, in which case the plants are classified as biennial (such as carrots and parsley). Many plants, comprising species with herbaceous, evergreen and woody growth forms, are perennial plants, which usually begin to form reproductive organs only after a few years and can live up to several thousand years [5]. Another classification of plants distinguishes between monocarpic species, which flower only once in their lifespan but can live up to almost 100 years [6] and polycarpic plants, which flower several times in their lifespan under appropriate conditions [4].

The ageing process in plants is called senescence and can include individual cells, organs or, ultimately, the entire plant [7,8]. The best known example is probably the loss of the leaves of deciduous trees, which first change colour and then fall off (abscise) every autumn for resource reallocation. Senescence is a genetically controlled degeneration process that is rapid and distinctive in monocarpic plants towards the end of their reproductive phase, whereas polycarpic plants undergo a more gradual decline that is less clearly attributable to physiological processes [4]. Age determination in plants can be performed via tree ring analysis, radiocarbon dating, growth form analysis, long-term monitoring or fire history analyses or via more modern analytical methods such as DNA fingerprinting or mutation analyses. For individual plants, an age of up to 5000 years has thus been assigned, whereby the oldest plants are all woody conifers [9]. Special cases involve plants that reproduce asexually with clonal reproduction [10,11], including community-sized individuals that have a lifespan of more than 40,000 years [8,9] and seeds that can maintain their viability for more than 2000 years [12]. Coalified conifer cones retain their hygroscopic capacity to open and close after more than 10 million years in the ground; however, it is important to note that this is already dead tissue [13].

Thus, a unitary period of time that can be defined as “longevity” does not exist for plants. However, the longevity of plant species encompasses their evolutionary adaptations that protect them in their ecological niches from undesirable environmental influences (e.g., predators, mechanical overload, extreme weather conditions) in order for them to grow and spread their organs of dispersal (namely seeds or spores) at least once during their lifespan, thereby ensuring the survival of their species. For perennial plants, the chances of propagation increase with each additional year of seed or spore dispersal. In summary, the longevity of a given plant species is closely related to its life cycle and the completion of dispersal with the highest possible probability of reproduction.

#### 1.1.2. Product Lifetime as a Longevity Measure in Artificial Material Systems

The product lifetime, sometimes also referred to as the lifespan, covers the period from the manufacture of a product or material system until it is discarded and encompasses the time when it is actively in use (service life), the time when it is potentially usable but not in use (dead storage) and the time when it is non-functional but not yet discarded [14]. The length of time that a product is used varies greatly and can be predetermined depending on whether it is a disposable, consumable or durable product. A longer lifespan is not always advantageous or desirable: because of rapid technological developments, many products (or their software) are so outdated before they fail that they are no longer competitive and are replaced regardless of their condition. For example, the lifespan of 85% of all smartphones in the UK was below five years back in the 1990s [15], with more recent data stating that 42% of Germans use their smartphones for less than two years [16]. Potential ways of keeping a product attractive and competitive and, thus, of extending its lifetime, include its ability to be upgraded, its variability or its product attachment capability, all of which conceive of products as being evolving systems rather than rigid ones [17,18]. However, limits to this exist, especially if completely new technologies become established (e.g., analogue–digital switchover). A prominent example is the evolution of sound carriers from phonograph records to cassettes to CDs to streaming solutions.

In addition to user-related demands, larger devices are typically subject to slow but continuous processes such as wear, fatigue or creep, making failure attributable to overcritical mechanical stresses increasingly likely [19]. A product or material system that loses its functionality or usability is referred to as experiencing obsolescence, which can be subdivided into four different types: Material obsolescence (poor performance of materials and components), functional obsolescence (attributable to increased demand for functionality), psychological obsolescence (attributable to the lack of adaptability to trends or consumer behaviour) and economic obsolescence (attributable to high maintenance or operating costs compared with that of a new purchase) [15,20]. In addition, repeated reports are published concerning the planned obsolescence of products by the manufacturer in order to limit their lifetime and thereby keep sales figures high. One of the best known examples is the manufacturer-imposed 1000 h limit on the life of incandescent light bulbs, even though they could last much longer [21,22].

Future trends, user behaviour and material developments are difficult to predict. We therefore focus in the following sections of this paper mainly on material obsolescence and the mechanisms that exist to extend the lifetime of products and material systems in this respect. Such considerations can have a particularly strong impact on the product life of a wide range of material systems used in industry and in kitchen appliances (customer satisfaction with these products is particularly low with regard to their lifetime) [15].

Assessment of the expected longevity of technical products therefore depends markedly on the intended use of the product and is determined by the customer’s expectations. Thus, to achieve longevity, a product does not have to last as long as possible, but rather to operate flawlessly up to its expected lifetime, before possibly continuing to operate through its reuse or repair or via its individual components after their disassembly in a circular economy.

### 1.2. Natural Material Cycles as Inspiration

In these times of energy and resource scarcity, a shift from linear to circular economy is being envisaged but is far from straightforward. Instead of mining raw materials, processing them into a human-made product and throwing them away at the end of their service time, society’s material flows should be closed through circular use of materials, components and products [23]. The inspirations for achieving this goal are natural closed loop systems (e.g., carbon cycle, sulfur cycle, nitrogen cycle, water cycle, phosphorus cycle, oxygen cycle), which, via the separation of materials and their reuse, have been prime models for clean material cycles through recycling and removing hazardous substances [24]. However, we can also learn from the long-term structural and mechanical integrity, i.e., the functionality, of biological systems, with regard to product-related value retention through reuse, repair, redistribution, re-manufacturing and refurbishment [25]. Thus, bio-inspired or biomimetic solutions for both clean material recycling and product-related value retention can contribute markedly to the global challenges described in Sustainable Development Goal 12 “Ensure sustainable consumption and production patterns” of the 2030 Agenda [26].

Although natural closed loop systems, in which nothing seems to be wasted and everything appears to be returned to the cycle, serve as prime models, studies of the life cycle of plants show that plant material has been deposited as landfill material under certain circumstances over millennia: exposure to heat and pressure within the Earth’s crust has changed such dead plant materials into coal, petroleum and natural gas, which are now classified as fossil fuels. The extraction and burning of these fossil fuels to provide energy for human needs releases carbon dioxide (CO${}_{2}$) directly into the Earth’s atmosphere, where it ultimately re-enters the carbon cycle. Since the Industrial Revolution (around 1750), the concentration of primary greenhouse gases in the atmosphere, which includes CO${}_{2}$, has increased significantly. This fragile equilibrium has been disturbed and a new equilibrium has being established with all the consequences of global warming. We have used the example of the carbon cycle to demonstrate the strong influence that humans have on nature and the ease with which an equilibrium can be destroyed. The unfortunate misunderstanding that nature “does not produce waste” clearly shows that a precise knowledge of natural models is a prerequisite for sustainable action.

### 1.3. Biological Material Systems as Inspiration

In the context of biomimetics [2], functional principles of living organisms can serve as models for lifetime-adapted structural integrity and the maintenance of functionality of human-made products. Since plants and products can be considered as material systems, a transfer of functional principles from biology to engineering is possible. Despite the interdisciplinary nature of this transfer between natural scientists (e.g., biologists, chemists, physicists), mathematicians, computer scientists and engineering scientists (e.g., material scientists, civil engineers, architects, designers), unambiguous communication is a particular challenge because each scientific discipline has its own technical language, different ways of thinking and working and subject-specific writing and symbol systems. However, also within a certain discipline, diverse understandings and definitions of technical terms may be found among different scientists, authorities or sub-fields. Therefore, we introduce here some of the key terms, so that, even in the case of supposedly simple concepts, the similarities and dissimilarities become clear and any overlaps and demarcations are addressed. Further definitions of terms are available in the glossary.

In this study, we focus on biological and artificial material systems (including conventional technical material systems and biomimetic material systems) because they exhibit chemical and physical properties and functions and functionalities that exceed those of the individual materials involved. Plants and technical products have in common that they are hierarchically structured. In plants, a few substances (e.g., pectins, cellulose, hemicellulose, proteins, lignin) form the cell walls of various tissues (e.g., parenchyma, collenchyma, sclerenchyma) occurring in the organs (e.g., root, stem, leaf, flower) of the plant body [27]. From a botanist’s point of view, the cell walls are material systems, as are the patterns of several tissues in the plant organs. In engineering, a distinction is made between individual materials (e.g., metals, polymers, ceramics) and material systems that are composed of several materials (e.g., fibre-reinforced composites, reinforced concrete), which can be part of a component [28]. Such an exact distinction is not possible in biology because the transitions between material and structure are so smooth that Wegst et al. [27] coined the term “structural materials”.

### 1.4. Material Systems Operate over Time

Interestingly, in biology, a tissue is defined as the interaction of similar cells that perform a specific function. Yet, what exactly is a function? This question is not easy to answer because there is no experiment that determines a function. In the past, many philosophers have addressed the issue of function and purpose. In Aristotle’s Philosophy of Nature, the concept of function lies within the end, purpose or “final cause” (télos) [29]. However, Aristotle (384–322 BC) had previously observed that télos does not necessarily involve deliberation, intention, consciousness or intelligence [30]. In Immanuel Kant’s philosophy (1724–1804), an object with intrinsic value can be regarded as an “end-in-itself” [31]. Ernst Mayr (1904–2005), a philosopher of biology, denies all teleological explanations and considers all living matter to be “an *a posteriori* product of natural selection” ([32], p. 1506). In engineering, the function of an object is determined from the beginning by the engineer. Here, we can cite the well known example of the construction of a table. Engineers decide that they will ask a carpenter (*causa efficiens*) to make a dining table (*causa finalis*) out of wood (*causa materialis*) and that it should have four legs and a rectangular table top (*causa formalis*). In biology, however, we have to attribute functions because we see the results of biological evolution. The function of a component is obtained by a functional analysis of the way in which the component is embedded in the system and what its contribution to system performance is [30]. The challenge of functional attribution in biology is beautifully summarised in the title “If bone is the answer, then what is the question?” [33].

Functionality, however, describes the quality of a function and is often expressed as a percentage between 100% and 0%. Elegantly, the percentage description of functionality applies to biological and artificial systems, so that they can be easily compared with and among each other. For example, the quality of self-healing of a plant organ or a self-healing artificial material can be calculated via the ratio of a selected mechanical property in various states such as the freshly wounded or the sealed or the healed state with reference to the intact state. Depending on the considered states, the available equations calculate a dimensionless value for “healing efficiency” [34,35,36], “wounding effect” or “healing effect” [37] or “repair efficiency” [38] or can even suggest the name of a respective property, such as “stiffness recovery ratio” [39] (cf. Section 3.2).

The selection of properties to be compared in different states [40], such as geometric properties (e.g., axial second moment of area [41], torsional second moment of area), mechanical properties (e.g., Young’s modulus [36], bending elastic modulus [41], torsional modulus), structural properties (e.g., flexural rigidity [39,41], torsional rigidity, tensile strength [36,42]) and other physical properties (e.g., roughness of fracture surface, air flow [38]) depends on the function to be determined.

This leads to the next point, namely that a function can be achieved through various functional principles. Using the example of the self-sealing function, we can see that during the course of biological evolution, a variety of underlying functional principles have been developed to seal wounds rapidly, depending on the body plan [43] of the respective plant [44]. Latex-bearing plants such as *Euphorbia tirucalli* or *Ficus benjamina* seal wounds through the release of latex that is stored under an over-pressure of up to 8 MPa in their laticifers [44,45]. Moreover, wounds of the succulent leaves of *Delosperma cooperi* are sealed within approximately 60 min by the functional principle “deformation of the entire leaf until the wound edges meet”. Hydraulic shrinking and swelling are the main driving forces and are sped up by growth-induced mechanical pre-stresses in the tissues [46,47,48,49]. The numerical model of Klein et al. [49] and the analytical model of Konrad et al. [48] have shown that each sealing principle can also individually lead to a complete closure of the wound. We can therefore interpret the self-sealing function of *Delosperma cooperi* as a redundant system (cf. Section 3.1).

### 1.5. Aim of the Work

At the interface between biology and technology, we have studied two concepts of the longevity of biological material systems, namely robustness and resilience, as inspiration for biomimetic material systems. These inspirations can be used to understand the way in which technical products can be better designed to achieve the service time expected by the consumer while retaining their functionality. According to our fundamental concept, we use functionality as a dimensionless measure to quantify the system function. Thus, we can represent both robustness and resilience as functionality of the entire system as a function of time, which allows us to make comparisons not only within biological material systems and biomimetic material systems, respectively, but also between them. We have studied robustness in the sense of fault tolerance or remaining unharmed with respect to the underlying principles damage resistance through safety factors, gradual transitions, response, acclimation, adaptation and optimisation. Furthermore, we studied resilience in the sense of failure tolerance or returning to an original state by means of the underlying principles redundancy and self-repair. In addition, we have compiled a glossary of the technical terms that are used in diverse contexts or disciplines with sometimes contradictory or overlapping meanings. The review including the glossary is intended as a source of inspiration and a guideline for interdisciplinary scientific teams developing biomimetic material systems with a high longevity potential.

## 2. Robustness

**Figure 2:**
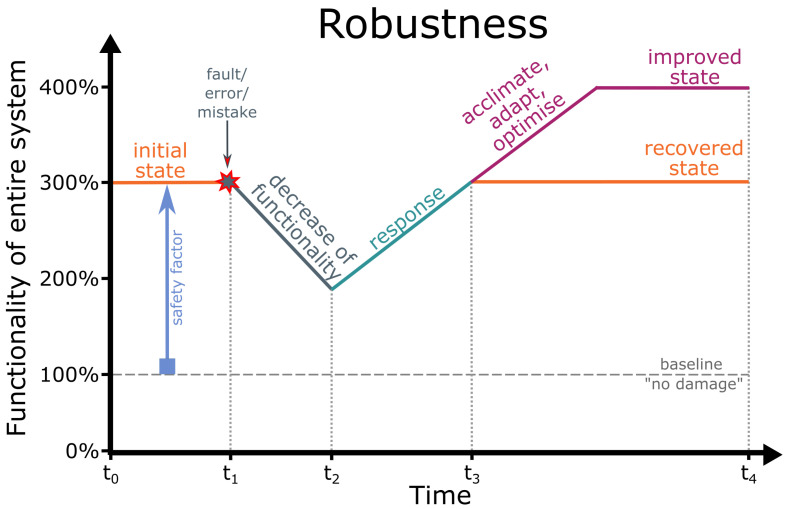
Robustness of biological and artificial material systems depicted as the functionality of the entire system as a function of time. At time $t_{0}$, the material system is in an initial state that is a multiple of 100% functionality (baseline “no damage”), resulting from the safety factor (blue arrow), composed of, for example, superimposed geometric, mechanical and structural gradients that make the material systems damage-resistant. Following a fault/error/mistake event at time $t_{1}$ (red star; e.g., multiple wind loads), the functionality of the material system first decreases (e.g., wind-induced change of plant configuration) and increases again at time $t_{2}$ via a rapid response (e.g., streamlining of the plant and its organs within seconds to minutes) and returns to a recovered state exhibiting initial functionality at time $t_{3}$ (e.g., recovery of plant configuration). However, the biological and artificial material systems can reach an improved state at time $t_{4}$ that exceeds the initial state and thus also the recovered state through the ability to react to environmental changes. Since the functionality never falls below the baseline of 100% functionality, the material system remains unharmed.

We have addressed the biology–technology interface in order to investigate robustness of functionality of material systems [50,51,52,53,54,55]. Robustness in terms of fault tolerance or damage prevention depends on the material system, the respective function and the type of perturbation [53,54]. Figure 2 provides a schematic drawing of the functionality of the entire system as a function of time, showing that robust material systems remain unharmed or are unaffected by most faults, errors or mistakes because they possess a safety factor that allows them to withstand multiples of stress and strain without marked damage. The safety factor of material systems results, for example, from superimposed geometric, mechanical and structural gradients that make them damage-resistant or from their ability to react to environmental changes without harm through response, acclimation, adaptation and optimisation.

We have addressed the biology–technology interface in order to investigate robustness of functionality of material systems [50,51,52,53,54,55]. Robustness in terms of fault tolerance or damage prevention depends on the material system, the respective function and the type of perturbation [53,54]. Figure 2 provides a schematic drawing of the functionality of the entire system as a function of time, showing that robust material systems remain unharmed or are unaffected by most [fault]faults, errors or mistakes because they possess a [chapter safety factor]safety factor that allows them to withstand multiples of stress and strain without marked damage. The safety factor of material systems results, for example, from superimposed geometric, mechanical and structural [chapter gradients]gradients that make them damage-resistant or from their ability to [chapter reaction]react to environmental changes without harm through response, acclimation, adaptation and optimisation.

Damage prevention is a technical standard today because engineers have to guarantee that the technical product will not break during normal operation or in the case of misuse. However, technical products that remain unharmed after damage are often “overbuilt”, which means high material consumption but also high “safety factors” and in particular “reserve factors” (cf. Section 2.1). With a focus on efficient use of natural resources and reduction of waste generation, the transfer of plant-inspired damage prevention to biomimetic products can contribute to the challenges described in the 2030 Agenda such as target 12.1 “By 2030, achieve the sustainable management and efficient use of natural resources” and target 12.5 “By 2030, substantially reduce waste generation through prevention, reduction, recycling and reuse” [26].

### 2.1. Safety Factor—The Sum of Individual Elements

The safety factor (*SF*), sometimes referred to as the “factor of safety” describes the relationship between the maximum load that a material, structure or material system can withstand and the stress to which it will be subjected under typical conditions [56]. For plants and buildings, for example, the safety factor describes the multiple by which the structures can carry more than their actual static load. The most common way to calculate the safety factor is given in Equation (1) and results in a dimensionless quantity. In some cases, however, a percentage value is stated, in which case the result is multiplied by 100%. In some disciplines, the “margin of safety” (*MoF*) is used instead, which is a derived value of the safety factor (Equation (2)). The *MoF* can be expressed as a relative value or as a percentage, although the latter is more common. In the building sector, the “reserve factor” (*RF*) must be calculated, which is usually the quotient between the calculated and the legally determined safety factor specified in the European standards (Equation (3)). To comply with the legal requirements, the “reserve factor” must be 1 or more (RF≥1). Since the equations and input variables used vary, it is important to carefully check what exactly was calculated. For a better comparability, we will refer to the dimensionless safety factor in the following.

(1) $$SF\;\left(/\right)=\frac{maximum\;load}{static\;load}$$

(2) MoF(%)=(SF−1)·100%

(3) $$RF\;\left(/\right)=\left(\frac{calculated\;SF}{legally\;determined\;SF}\right)$$

With a safety factor of SF=1.0, a structure can support itself but no further loads. Any increase in this value indicates that the structure can also withstand higher loads (e.g., attributable to external environmental influences such as wind or snow). As soon as the safety factor falls below 1.0, the system is no longer stable even under its own weight.

The use of the safety factor in artificial material systems is as old as the Industrial Revolution and was applied as early as the 1860s by a German railway engineer who used a factor of 2 to protect the tracks from failure under tensile load [57]. Since then, the calculations of the safety factor have become increasingly sophisticated and the following five factors are considered for this calculation: (1) the occurrence of larger forces than expected; (2) poorer material properties than anticipated; (3) calculation errors in the failure theory; (4) possible unknown failure mechanisms; and (5) human mistakes in design [58]. Depending on the types of loading that occur, a single safety factor value might not be sufficient for a material or a material system; for example, one value for strength and one value for fatigue should be considered [58].

In addition to the safety factor, the probabilistic risk assessment method has gained increasing importance, since the end of the last century, as a safety assessment tool for material systems and structures. In this approach, the probability of failure, together with the magnitude (or severity) of the possible consequences are taken into account [59]. However, the probabilistic risk assessment should not compete with the calculation of a safety factor but should provide additional information for the design process [60]. Duncan’s work [61] on safety factors for geotechnical engineering practices incorporates probability scenarios and a cost factor. When the most likely scenarios are assumed, a safety factor of 1.5 is sufficient to protect a building from sliding on sand. Depending on the nature of the soil and the materials used, one should add uncertainties of various magnitudes to this value; for example, a standard deviation σ of 0.15 should be considered to take into account the friction angle between the building and the sand, thus resulting in a lower limit of the safety factor of 1.35 and an upper limit of 1.65. To obtain a higher certainty, which covers about 99.7% of all cases, one should apply the three Sigma rule. In the presented example, this would result in certainty factors of between 1.05 and 1.95. If a failure of the structure should not be "catastrophic", an estimation of the additional costs can be used to decide on the number of standard deviations to apply [61].

**Figure 3:**
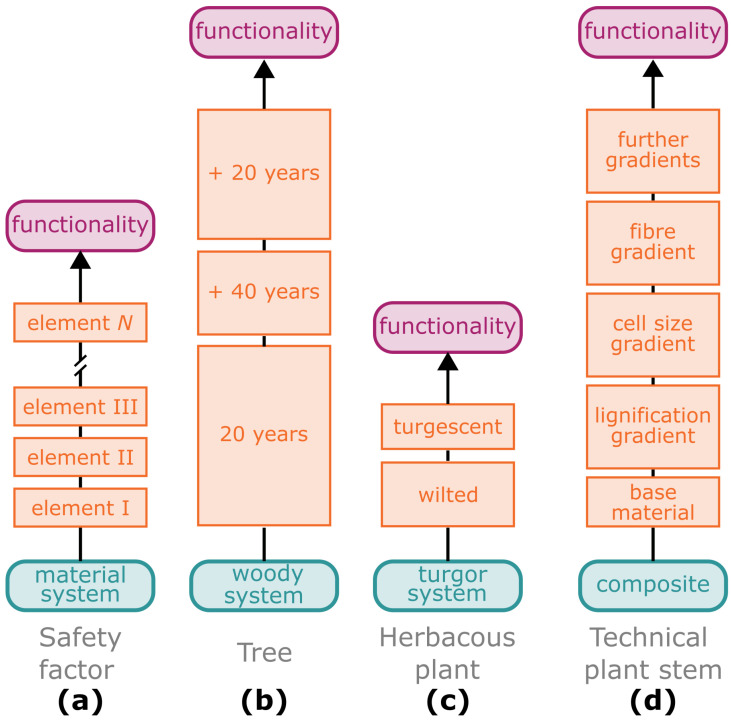
Safety factor and contributing elements of various systems. (**a**) Similar to a series circuit, the safety factor is the sum of individual elements contributing to the overall functionality of a material system. (**b**) As *Populus tremuloides* is a tree, we define its functionality as the safety factor of its trunk, a factor that depends on secondary growth. The safety factor increases on average from 2.4 for 20-year-old trees to 5.1 for 80-year-old trees [64]. (**c**) Since *Gerbera jamesonii* is an herbaceous plant, we define its functionality as the turgor-dependent safety factor of its peduncle. The safety factor is 0.95 for wilted peduncles. On increasing the turgescence of the tissues, the safety factor increases to 1.42 [65]. (**d**) Since the technical plant stem is a biomimetic lightweight construction, its functionality is defined as mechanical performance in relation to sample weight. The example of the technical plant stem (cf. Section 2.2) illustrates that the safety factor markedly increases by adding up various gradients.

In plant biomechanics, the safety factor for herbaceous plants or trees is calculated to indicate the multiple of their own weight that they can withstand under additional dynamic loads (Figure 3). Niklas [62] has been able to show, by his work on peduncles (stalks supporting the flower or inflorescences) of garlic (*Allium sativum*) that their safety factor of about 1.85 is similar to that of technically used wooden columns and that this value is strongly dependent on the external influences to which the plants are exposed during growth (see also Section 2.3.3 on acclimation). The safety factor of plants that were grown outdoors but in a protected plastic enclosures with an open top was measured at 1.29 and that of plants grown under fully controlled conditions in a greenhouse even only at 1.11. Even larger values ranging from 1.7 to 2.9 were found by Langer et al. [63] for the petioles of herbaceous plant leaves. The last mentioned values can be explained on the basis that the petioles not only have to carry the static load of the petiole and the lamina, whereby the latter has a considerably larger cross-sectional area than the petioles themselves, but also have to withstand additional loads such as wind gusts, rain, snow and perching animals. Langer et al. [63] calculated the dimensionless safety factor of the petioles as a ratio of the critical length ($l_{max}$) and the real length ($l_{real}$). They presented two cases for the calculation of the critical length ($l_{max}$): (1) for peltate leaves in which the lamina is arranged like a top load on the petiole; (2) for foliated leaves in which the petiole is situated at the margin of the lamina. King and Loucks [64] calculated the gravitational safety factor ($g_{max}/g$) for American quaking aspen trees (*Populus tremuloides*) and found a safety factor of 2.4 for 20-year-old trees (Figure 3b). Interestingly, the safety factor increased with the age and secondary growth of the trees, with 80-year-old trees that were mechanically “overbuilt” showing a safety factor of 5.1. However, what happens when the safety factor falls below the critical value of 1.0? In this case, the plant can no longer support its own weight, an observation that is probably familiar to most of us from the wilting of cut flowers in vases. This well known phenomenon was analysed with respect to the safety factor of *Gerbera jamesonii* peduncles (Figure 3c). The safety factor was found to be 1.42 for fresh, fully turgescent plants, whereas in wilted plants with drooping flower heads, it decreased to 0.95 within 24 h [65].

### 2.2. Damage Resistance through Multiple Gradients

**Figure 4:**
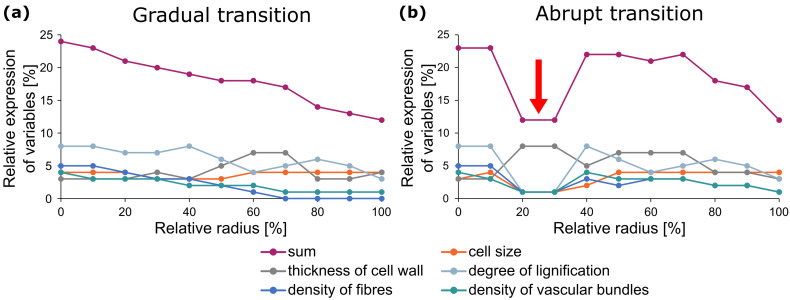
Types of transitions in plant structures. The radius of a cross-sectional axis is given as percent and the various properties are shown as relative values to allow the calculation of a sum (purple dots and line). The sum of various influencing properties shows either a transition (**a**) with gradual changes or (**b**) with an abrupt change (red arrow).

Generally, gradients create smooth transitions between major changes in geometric, mechanical and structural properties of material systems. Often, these smooth transitions are protected against damage by several superimposed gradients [66]. Their opposites, namely abrupt transitions, represent predetermined breaking points. These gradual or abrupt transitions can be found on various hierarchical levels in both biological and artificial material systems (cf. Section 1.3) resulting in a complex system [51]. However, the total of all gradual and/or sudden changes of influencing variables determines whether the transition is smooth or abrupt (Figure 4). In plant leaves, on the one hand, we find superimposed gradual transitions resulting in a damage-resistant transition zone between petiole and leaf lamina [63,67,68,69]. Moreover, we find abrupt transitions leading to a spatio-temporal controlled shedding of individual plant organs (abscission), such as between branches and petioles resulting in leaf fall in autumn [70,71]. Another example is the chain-like arrangement of branches in certain cacti (*Opuntioideae*), in which shedding enables vegetative reproduction [10,11,72]. The concept of shedding plant organs (abscission [70] and autotomy [73]) and discarding animal appendages (autotomy [74]) is discussed as inspiration for future smart structures with controlled failures occurring in predefined positions in the structural scheme. Possible breaking points are construction joints, deliberately weak zones, specially designed reinforcement bar configurations and fuse-type elements [55].

**Figure 5:**
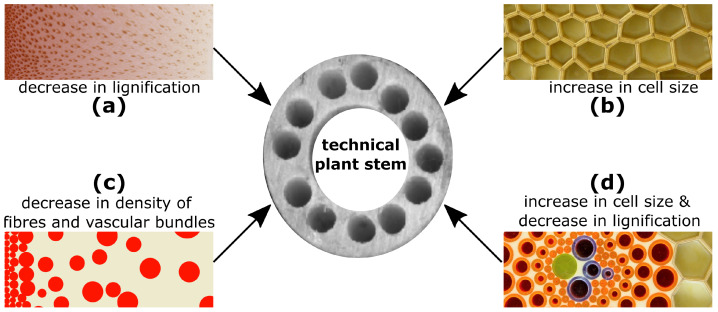
Technical plant stem with various embedded gradients. The technical plant stem (middle), which consists of glass fibres embedded in a solid matrix of epoxy–vinyl–ester, was produced by a braid-pultrusion technique. It was inspired by multiple gradients including those from the stem wall of *Arundo donax*. In a radial direction from the periphery towards the centre, we find (**a**) a decrease in lignification of the parenchyma cell walls, (**b**) an increase in cell size of the parenchyma and (**c**) a decrease in the fibres and vascular bundles (red) embedded in the parenchyma (white). In addition, (**d**) the sclerenchmya fibres (orange) of the vascular bundles (xylem in blue, phloem in green) show an increase in cell size and a decrease in lignification towards the parenchyma cells (brown).

A prime example of a biological damage-resistant structure is the hollow stem of the giant reed (*Arundo donax*) [75], whose multiple gradients at various hierarchical levels have served as the inspiration for the so-called “technical plant stem” [76] (Figure 5). The giant reed is up to 6 m high and grows in dense stands. No known observations have been made of *Arundo donax* stems showing significant damage in the field, even under additional stresses such as wind gusts, snow loads or perching animals (O.S.; information from personal interviews). The slender upright and slightly tapered stems are divided into internodes and nodes. The stem walls of the internodes exhibit several gradients, which meet all theoretical considerations and needs for a composite material optimised to withstand dynamic bending loads [76]. The load-bearing tissues, such as the highly lignified parenchyma (Figure 5a) and the lignified fibres and vascular bundles (Figure 5c,d), are concentrated in the periphery with a gradual reduction in cell wall thickness and an increase in cell size of the parenchymatous tissue (Figure 5b). Moreover, the degree of lignification of the parenchyma (Figure 5a) and the density of the strengthening tissues (Figure 5c) gradually decreases in a radial direction from the outside towards the centre. The vascular tissues, together with the fibres, are distributed in the parenchyma, an arrangement that can be interpreted as an adaptation to an optimised damping of the oscillations caused by wind loads or by passing animals [77]. The gradual decrease in lignification between the cells of the vascular tissues and the parenchyma (Figure 5d) can be interpreted as an adaptation to avoid delamination between stiff and less-stiff tissues [78].

These gradients (cf. Figure 3d) together with additional inspiration for lightweight construction from other plant structures have, as mentioned above, provided inspiration for the “technical plant stem”, such as that produced by means of a braid-pultrusion technique. Various fibre bundles can be incorporated into the technical plant stem in both axis-parallel and diagonal directions in a wide variety of matrices. Diagonal fibre bundles provide high torsional stiffness and toughness, high vibration damping and excellent structural integrity. Axis-parallel fibre bundles exhibit additional high tensile stiffness and toughness. By varying the density, arrangement and angle of the fibres in the respective layers, “technical plant stems” can be created with high robustness and can be targeted for particular loading situations [79]. For example, the technical plant stem shown in the middle of Figure 5 consists of glass fibres and a solid matrix of epoxy–vinyl–ester and has a density of 1850 kg m^−3^. It exhibits a logarithmic decrement of 0.084, which is a measure for its damping functionality. In addition, this technical plant stem has withstood the impact of a 50 J pendulum with a maximum force of 60 kN (Charpy impact test) without significant damage (notched impact strength >325 kJ m^−2^, absorbed impact energy >50 J) (unpublished data, by courtesy of T. Speck, University of Freiburg, Germany).

### 2.3. Damage Resistance through Reactions to Environmental Changes

The terms “response”, “acclimation” and “adaptation” from biology and “stimulus-responsive”, “adaptive” and “intelligent” or “optimised” from materials science and engineering present a particular challenge when it comes to finding inherent similarities or dissimilarities. In the field of biomimetics, confusion in the use of such terms becomes especially problematic when scientists claim that functional principles of biological models can be transferred to technical applications and, thus, that a biological model and a biomimetic product have the same function. These uncertainties are not limited to the various scientific disciplines, but are also related to the terms having various meanings at different hierarchical levels, from materials to material systems to entire plants or technical components. On closer inspection, the confusion is particularly striking with regard to the terms “stimulus-responsive” and “adaptive” in the field of materials science. In the following, we will show that plants and their material systems and human-made materials and components can gain robustness if they can react structurally and mechanically to withstand higher environmental stresses without major damage.

Unlike animals, most plants are immobile and firmly tied to their location. Thus, they are exposed to changing environmental conditions at all times. In unfavourable environmental conditions such as storms, drought, heat or cold, plants cannot run for cover but must react *in situ* in order to survive. We discriminate between short- and medium-term reactions of individual plants and long-term reactions of entire plant populations [80,81]. Table 1 provides an overview of plant reactions with a focus on mechanical stimuli such as wind loads, rain drops or contact by passing animals.

In analogy to the reactions of plants given in Table 1, we present Table 2, which contains corresponding technological terms and biomimetic examples, where available.

#### 2.3.1. Wind-Induced Response of Plants and Plant Organs

A prime example for damage prevention in the plant kingdom is the reconfiguration of stems, branches and leaves of individual plants in response to wind loads. Reconfiguration by means of streamlining within seconds and minutes reduces the projected surface area and, consequently, the resulting drag force. A simple measure for the effect of drag reduction by reconfiguration is the Vogel exponent V, which is a dimensionless number such that the power law with the wind speed *u* reads: $Df\;\propto \;u^{2+V}$ [82]. The Vogel exponent V can be calculated as the slope of a double-logarithmic plot of the velocity-specific drag as a function of velocity [83]. Experimental data from various plants (e.g., grasses, reeds, trees, algae), plant organs (e.g., leaves, flowers) and fluids (e.g., wind, water) reveal that their Vogel exponent varies between −0.2 and −1.2. In the range of the flow speed faced by plants, the Vogel exponent has a typical value of −1.0 [82]. Field experiments on six-meter-high individual plants of *Arundo donax* have shown that the value of the Vogel exponent V and thus the reduction of the drag force *D_f_ (u)*, is a function of the wind speed *u* [84]. At low wind speeds of up to 1.0 m s^−1^, the Vogel exponent V = −0.12, resulting in $Df\;\propto \;u^{1.88}$. For higher wind speeds between 1.5 m s^−1^ and 10 m s^−1^, the Vogel exponent V = −0.71, leading to $Df\;\propto \;u^{1.29}$. The difference in drag force results from increasing streamlining at high wind speeds. If we compare the measured drag force with streamlining at high wind speeds with the calculated drag force without streamlining by extrapolating the relationship at low wind speeds, we find a percentage drag reduction of 46% at a wind speed of 4.0 m s^−1^ and 73% at 10 m s^−1^ [84]. Thus, streamlined plants can withstand markedly higher wind loads without serious damage.

#### 2.3.2. Stimulus-Responsive Biomimetic Applications

However, damage prevention by drag reduction through streamlining is limited for engineering applications for various reasons. State-of-the-art technical constructions often consist of various rigid elements connected with hinges and, as, for example, in the case of an umbrella, additionally with a flexible membrane. By restructuring the individual elements, the overall shape of the system can be streamlined. The example of an umbrella shows the limits of the change to a streamlined shape. An everyday experience is that a gust of wind is enough to deform the frame of an umbrella in such a way that it can never return to its initial state. Therefore, if the wind is too strong, we take the precaution of closing the umbrella before the frame breaks. Now the umbrella is in a streamlined shape, but its functionality has dropped to 0%. Thus, an umbrella construction is not robust with respect to strong wind loads.

Even with a biomimetic approach [2], i.e., learning from the biological model, limitations cannot be overcome if the framework conditions are markedly different. A popular example in textbooks is the comparison of the constructions of a grass stalk and a tall tower. However, it is not the different size dimensions (centimetre vs. meter) that are crucial; the limitation lies in the amplitude of the damped oscillation of the grass stalk after a wind gust. These amplitudes cause no problems for plants, whereas meter-large amplitudes at the tower apex are unacceptable for people who live, work or eat in the tower. Therefore, streamlining in the wind by bending is not an option that can be considered for buildings. Slender structures are known to be highly sensitive and susceptible to wind-induced motion. In this context, engineers aim to tailor the external form of tall buildings in order to create an aerodynamic shape that minimises wind loads and mitigates associated structural motions. Alternatively, engineers can in future create tall buildings with a dynamic facade that can morph to the changing complex wind environment in urban areas. Ding and Kareem [91] have presented the concept of autonomously morphing wind-resistant structures. They claim that building topologies of the future must have dynamic facades that actively adjust their profiles to counteract the effects of changing wind conditions.

In recent years, various systems inspired by the responsive material systems of plants, which are hinge-less structures that can deform in their entirety, have been developed [92]. Plant-inspired architectural projects for responsive buildings and building envelopes include the *Flectofin*, a mechano-responsive facade shading system derived from the compliant pollination system of the flower of the bird-of-paradise plant (*Strelitzia reginae*) [93]; the *Flectofold*, a mechano-responsive facade shading system inspired by the hinge-less motion of the underwater snap-trap of the carnivorous waterwheel plant (*Aldrovanda vesiculosa*) [94]; the 14 m-high *Urbach Tower* [95], a humidity-responsive and self-shaping timber tower; and the *HygroSkin* [96], a humidity-responsive building envelope inspired by the humidity-driven motion of spruce cones.

#### 2.3.3. Mechano-Stimulated Acclimation of Plants

Since plants can perceive external mechanical stress [85], they are able to acclimate to wind, rain and perching animals. The first studies concerning the wind-induced acclimation of trees were published in 1803 by Knight [86]. In 1973, Jaffé defined mechano-stimulated adjustments of plants as thigmomorphogenesis [87], which is an alteration of growth pattern by changes in gene expression [88]. Although the changes depend on the plant species and on the type, duration and frequency of the stimulus, thigmomorphogenetic effects are mostly represented by changes in plant allometry. Wind-affected trees exhibit reduced shoot elongation together with an increased radial growth, resulting in a shorter but thicker trunk that can withstand higher mechanical stresses without undergoing significant damage. In addition to morphological changes caused by altered allometry, acclimation can also be manifested in anatomical alterations, starting at the cellular level and extending to the whole plant including changes in the mechanical properties of plant tissues [81,89]. All these changes add up to a substantial contribution to the safety factor of plants. Thus, the thigmomorphogenetic effects of “trained” trees and herbaceous plants are the result of the physiological growth changes [88] of individual organisms and, unlike biological adaptation, cannot be inherited by the next generation.

#### 2.3.4. “Trained” Plant Material Systems

**Figure 6:**
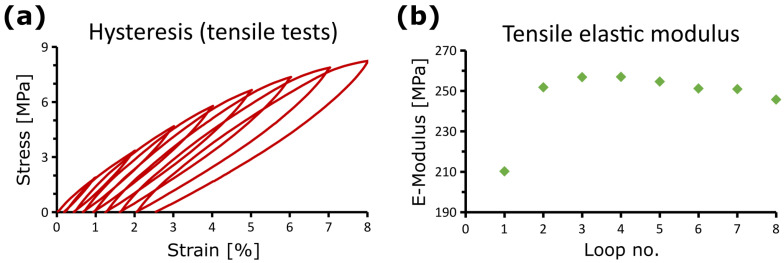
Results from a hysteresis experiment of a biological material system (rhizome samples of *Arundo donax*). (**a**) Eight hysteresis curves with increasing tensile strain (in 1% strain steps). (**b**) The tensile elastic modulus changes with respect to the repeated and increasing strain. The first loop reveals a markedly lower modulus compared with the subsequent loops.

The effect of multiple repeated wind gusts on plant material systems can be studied in cycling tests. Figure 6 presents the changes in the tensile elastic modulus over the course of eight cycle loops with increasing tensile strain (in 1% strain steps). An example is the mechanical performance of rhizome samples of *Arundo donax*, which can be regarded as a fibre-reinforced composite consisting of stiff fibres and vascular bundles embedded in a matrix of parenchyma (cf. Figure 5c). Since the “training effect” of the plant material system occurs within minutes, it is likely to be a response to the mechanical stresses in the form of an alignment of the tissues and their components and a viscoelastic-plastic behaviour of the biological material systems [105,106] rather than an acclimation based on altered growth patterns by changed gene expression. Interestingly, similar curves to those shown for biological material systems in Figure 6 can be obtained with artificial materials. In materials sciences, these artificial materials trained by cycle loops are known as “adaptive materials” [97,98].

#### 2.3.5. “Trained” Artificial Materials

Artificial materials are awarded the attribute “adaptive” if they can independently adjust to changing environmental conditions. The question, however, arises as to what extent adaptive materials differ from stimulus-responsive materials. According to Walther [97], a distinction can be made with respect to their management of and navigation within complex energy landscapes. Stimulus-responsive soft material systems alternate between two low energy states. The switching process by a trigger and counter-trigger is reversible. Even additional switching always induces the same change [97]. This is in contrast to adaptive material systems, which can dynamically adapt or evolve to new states under out-of-equilibrium conditions [97,98]. After a certain number of trigger/counter-trigger cycles, the material system stops responding to the same signal, adopts a distinctly different functional state or undergoes a change in properties. Examples for the training of artificial and biological material systems are strain hardening under inelastic deformation and hysteretic memory under cyclic loading. Conversely, this means that artificial material systems can be systematically and targeted “trained” depending on the strength, duration, frequency or number of cycling loops. However, a previously altered energy landscape and thus a memory function of “trained” material systems might no longer be present if the stimulus is absent or falls below a certain threshold. Relaxation is an example for the “forgetting” of the adjusted properties and thus the “trained” energy landscape [97].

#### 2.3.6. Adaptation and Optimisation

Following the example of biological evolution, evolutionary algorithms were developed in the 1960s. As sub-classes of the evolutionary algorithms, genetic algorithms and evolutionary programming were developed independently in the USA, whereas evolutionary strategies were developed by Schwefel [99] and Rechenberg [100] in Germany [101]. Evolutionary algorithms are biomimetic optimisation methods that involve the evolutionary principles of variation (= mutation and recombination) and selection (Table 3). These principles are executed iteratively in a loop, whereby new and optimised solutions (= offspring) are generated with each new generation and thus allow to find an optimal solution without knowledge of it beforehand. Thereby, evolutionary algorithms can be used universally and can even be applied to problems that cannot be formulated mathematically. On the one hand, the bird-like wing tips for aeroplanes and the design of a truss bridge or of an optical lens can be optimised by objective selection [102]. On the other hand, the subjective selection of coffee testers can be used to determine the best blend ratio of coffees to achieve a specific coffee taste [103]. Evolutionary strategy with the so-called comma strategy, i.e., only the offspring compete with each other (see also below; [104]), are particularly suitable for simulations and for applications using subjective quality evaluations [101].

**Figure 7:**
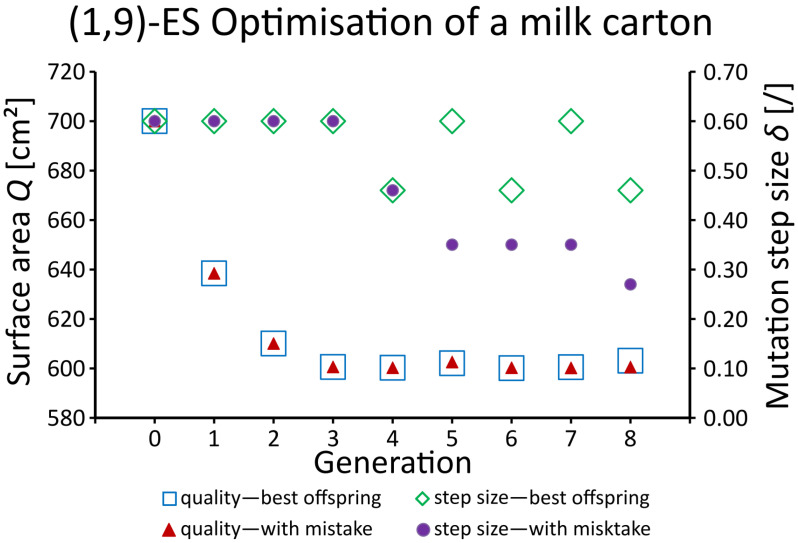
Results of milk carton optimisation with the (1,9)-Evolutionary Strategy (individual solutions). The goal is to package one litre of milk with the lowest possible material consumption, which can be objectively measured as the surface area *Q* of the carton. The parent individual started with $a_{P}=5$ cm, $b_{P}=10$ cm and $c_{P}=20$ cm and thus $Q_{P}=700$ cm${}^{2}$ and a parental mutation step size of $\delta_{P}=0.6$. The variation of the parental mutation step size of $\xi_{P}=const.=0.77$ results in a decrease in the step size of each offspring by $\delta_{O}=\delta_{P}\cdot 0.77$. Comparative results are shown here when the best offspring is always selected or when a mistake was made in generation 5 and only the second best offspring is accidentally selected. Despite this mistake, the optimisation with the evolutionary strategy is robust and approaches a minimum of material consumption.

As an example, we will describe the way in which the material consumption of a milk carton used for the packaging of one litre of milk can be minimised with the help of evolution algorithms (a detailed description can be found in [104]). The (1,9)-ES Optimisation presented in Figure 7 uses the comma strategy; namely, in each generation one parent individual produces nine offspring individuals, the best of which then becomes the parent of the next generation. During initialisation, we set the object parameters (= side lengths *a*, *b*, *c*) with a·b·c=1000 cm${}^{3}=1000$ mL and a,b,c>0 cm. In addition, we set the parental mutation step size $\delta_{P}$ and the variation of the parental mutation step size ξ. Since the mutation step size of the offspring is calculated as $\delta_{O}=\delta_{P}\cdot \xi$, the mutation step size $\delta_{O}$ decreases if ξ<1.0, increases if ξ>1.0 and remains unchanged if ξ=1.0. The optimisation quality Q=2(a·b+a·c+b·c) is the surface area of the milk carton. The goal is to minimise, by using the evolutionary strategy, the material consumption *Q* for a milk carton that will contain one litre of milk. By selecting the best offspring, i.e., the one with the smallest surface area *Q*, as the parent of the next generation, we have created a total of eight generations (Figure 7). In one run, we always selected the best offspring (blue rectangles). In another run, we made a mistake in generation 5 and did not select the best, but the second-best offspring (red triangles). The fact that the two quality curves are almost identical shows that the evolutionary strategy is robust enough to converge to a minimum surface area.

In general, evolutionary algorithms are optimisation methods that are highly robust. Even if the best offspring is not always selected (e.g., faults in hardware, errors in software, mistakes made by human beings), the algorithms ultimately lead to the optimal solution.

## 3. Resilience

**Figure 8:**
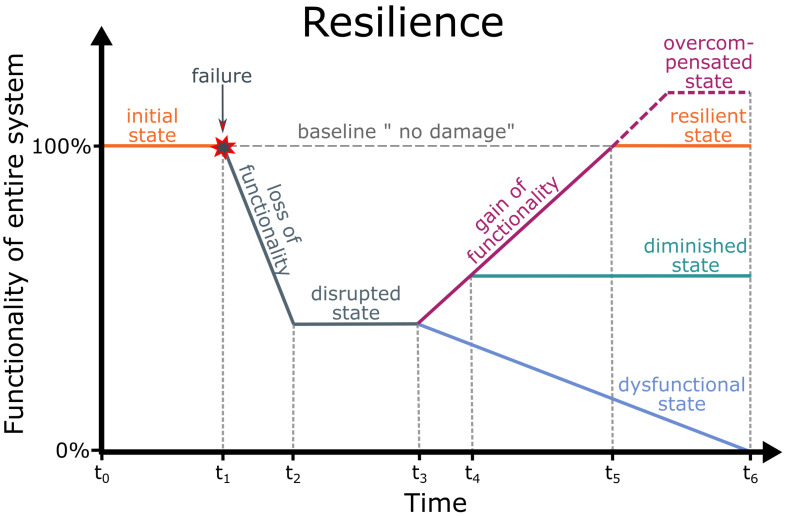
Resilience of biological and artificial material systems defined as the functionality of the entire system as a function of time. The material system starts with an initial state of 100% functionality (= baseline “no damage”) at time $t_{0}$. After a failure event at time $t_{1}$ (red star; e.g., damage), the material system continuously loses its functionality until it is in a disrupted state at time $t_{2}$. Starting at time $t_{3}$, four scenarios are possible: (1) the loss of functionality progresses until the entire material system is in a dysfunctional state (= 0% functionality; e.g., breakage, abscission of plant organs), (2) the material system regains part of its functionality and remains in a diminished state at time $t_{4}$ (e.g., incomplete damage repair), (3) the material system returns to the baseline exhibiting the resilient state with 100% functionality at time $t_{5}$ (e.g., complete damage repair) or (4) the effect of the failure is sometimes overcompensated and the final state is above 100 % functionality at time $t_{6}$ (e.g., lignification, scar or callus formation).

At the interface between biology and technology, we have studied resilience of functionality of material systems in the sense of failure tolerance or damage management [50,107]. In the event of failure, resilient material systems can, at best, return to their original state or even overcompensate their initial state (Figure 8). Resilient material systems have, for example, various concepts of redundancy that can compensate for the failure of individual elements or can self-repair damage in terms of rapid self-sealing and subsequent self-healing. In our understanding, resilience is not a correlate of robustness [54].

With a focus on sustainable development through the efficient use of natural resources and the reduction of waste generation, damage management can also contribute to the targets 12.1 and 12.5. of SDG 12 “Ensure sustainable consumption and production patterns” in the 2030 Agenda [26]. For us, it is often not acceptable that technical products remain in a dysfunctional or reduced functional state after damage. Instead, they should return to their initial state as quickly as possible. A rapid return to the resilient state through redundancy is widespread in technical products, while artificial material systems with self-repair function are rarely found. The plant-inspired abscission of organs, such as leaf fall in autumn, can serve as an example of a predetermined breaking point to fragment the product for reuse and to disintegrate its materials for recycling.

### 3.1. Resilience through Redundancy

**Figure 9:**
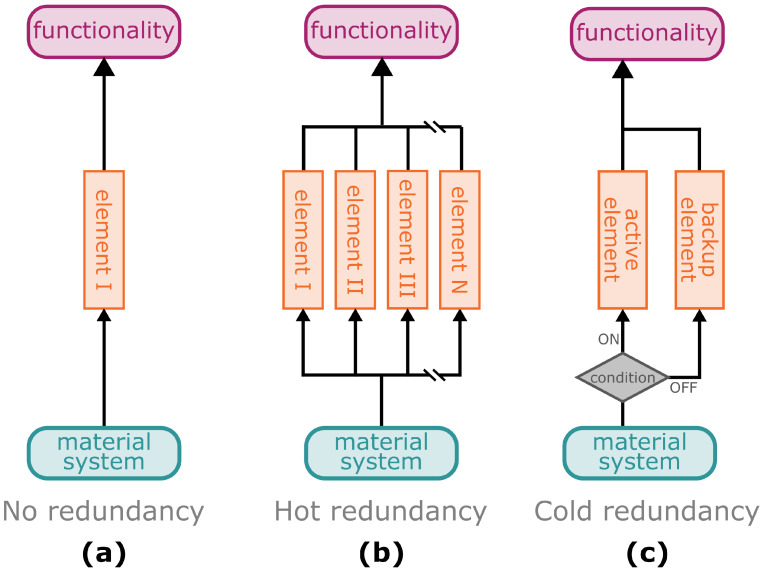
Various redundancy concepts. (**a**) If no redundancy is present, the functionality of the system is lost in cases involving element failures. (**b**) In a system with hot redundancy, several elements are involved in providing the functionality of the overall system. If one of these elements fails, the other elements can take over its function resulting in little or no loss of overall functionality. (**c**) In a cold redundancy system, at least one backup element takes over the function of an active element in the event of its failure. The overall system therefore does not lose its functionality or, at least, the downtime is substantially reduced.

Redundancy mechanisms increase the resilience of systems by maintaining their functionality, even in the event of failure of a single component or multiple components. Thereby, the functionality of a redundancy system can be compared with a parallel circuit of an electrical network, in which the individual elements operate independently of each other at the same time. In the case of the safety factor, in contrast, the individual elements (e.g., various gradients) are structured sequentially, which is similar to an electrical series circuit. If such redundancy does not exist, the failure of a single (sub-)element will cause failure of the entire system (Figure 9a). In technical or information technology systems, a distinction is made between hot redundancy (Figure 9b) and cold redundancy (Figure 9c) [108].

**Figure 10:**
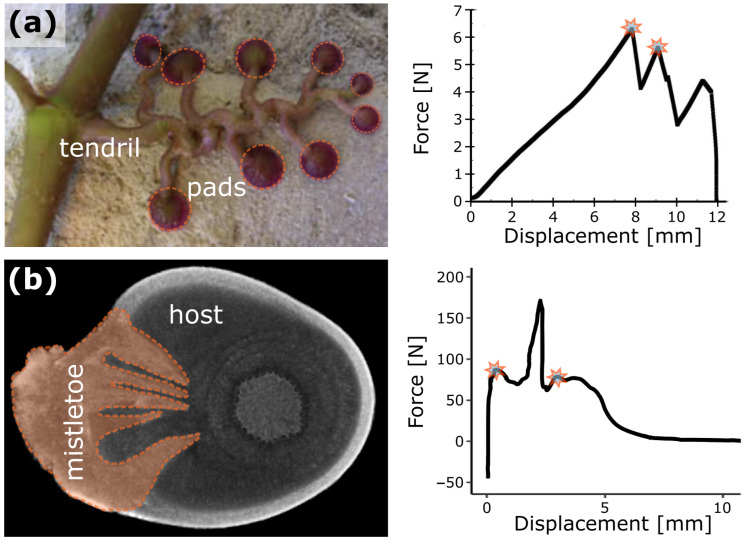
Hot redundancy mechanisms of biological material systems. (**a**) Adhesive tendril of Boston ivy (*Parthenocissus tricuspidata*) with nine redundant adhesive pads (circled in orange). The exemplary force-displacement curve shows stabilisation of the overall system after failure of individual pads (orange stars). Adapted with permission from [109]. (**b**) Cross-section of a micro-computed tomography image through the attachment site of a young mistletoe (*Viscum album*; coloured in transparent orange) and its host branch. Five wedge-shaped sinkers form the redundant mechanical and physiological connection between the two species. A force-displacement curve of a mistletoe–host sample under tensile load shows pre- and post-failure events, indicating the failure of individual sinkers (orange stars). Adapted from [110].

In a hot redundancy system, multiple elements are permanently involved in ensuring the functionality of the overall system. In the case of a failure of one or more of these subsystems, the remaining elements take over the failed functionality (Figure 9b). This type of system can be found, for example, in computer networks whose processing power is provided by several sub-units or in the energy supply system of critical infrastructure or industrial plants [108]. However, hot redundancy can also be found in plants and their mechanical anchoring structures. In the climbing plant called Boston ivy (*Parthenocissus tricuspidata*), several adhesion pads are found on a spring-like tendril and adhere to the substrate by means of an adhesive fluid. Mechanical pull-off tests have revealed that the overall structure still provides anchorage for vertical growth, even when individual pads lose contact and fail, as indicated by multiple force peaks representing individual failure events in the force-displacement curves (Figure 10a) [109]. The hemiparasitic European mistletoe (*Viscum album*) uses, especially in the first years of its combined growth with the host, several wedge-shaped sinkers (structures of the so-called haustorium, a modified organ common to all plant parasites) mechanically to anchor itself in the host and to form a physiological connection for water and nutrient uptake [110]. Under mechanical loading, the initial failure of individual sinkers occurs before complete failure of the entire connection is observed, which is likewise indicated by pre- and post-failure events in the respective force-displacement curves (Figure 10b) [111].

In both biological examples described above, the failure of single redundant elements (attachment pad or sinker) leads to the lost functionality being covered by the remaining elements. This allows the plants to compensate for the losses by growing new tissues or organs in the following growth period. However, this applies only up to a certain level, at which the individual failures accumulate to such an extent that system functionality falls below a critical threshold, leading to the failure of the entire structure.

In the case of cold redundancy, the system requires a built-in or external sensor unit that, in the event of a failure of primary elements, activates previously inactive (standby) elements, which take over the function and restore the functionality of the system (Figure 9c) [112]. A well known example that has been helping to save lives for decades is the reserve parachute in skydiving, which can be opened manually or automatically in the case of the failure of the main parachute. Cold redundancy in biology is found, for example, in facultative plant parasites. Plant parasites in general use the haustorium [113] to form a mechanical and physiological connection with their host and to draw water and nutrients from it. In the case of holoparasites that are not themselves photosynthetically active, carbon is also taken up from the host. Unlike obligate parasites, which are not viable without a host, facultative parasites can live and grow for at least a part of their lifecycle autotrophically without contact with a host [114,115]. These plants have a cold redundancy system of their physiological activity that, in the case of the loss of the primary supply from the host (e.g., host death), allows them to transition to host-free life and growth, even though this may be accompanied by reduced physiological activity. If their physiological functionality does not fall below a critical threshold, this “backup” energy harvesting system allows them to remain alive, either to form reproductive organs or to establish physiological and mechanical connections with a new host. However, the extent of host-free growth has often been analysed under laboratory conditions and is considered controversial among experts [115,116].

The cold redundancy mechanisms of the adhesion disc of the remora, also known as the suckerfish (*Echeneis naucrates*), enables adhesion generation (1) underwater, (2) to porous structures and (3) in the air. This is accomplished by compartmentalisation carried out by lamellae within the disc [117,118]. The remora can attach to fast-moving marine hosts as a “hitchhiker” to save energy. Some of the hosts, such as dolphins, can jump out of the water in an attempt to rid themselves of these unpleasant passengers [119]. In order to maintain adhesion during the water–air transition, the remora is capable of rearranging its lamellar compartmentalisation to prevent it from dropping off from its host. This redundancy mechanism has been translated by Li et al. [118] into a biomimetic flying robot that can perform rapid attachment and detachment operations both underwater and in air and can thus be used as a hitchhiking robot for exploration and transport in versatile terrains.

In addition to the structural-morphological and physiological redundancy described above, redundant structures in the plant kingdom are also found at the genetic level, whereby gene duplication can compensate for possible mutations or prevent the expression of new attributes [120,121] and at an ecological level, whereby redundancy can ensure the functioning of ecosystems in the case of events that lead to temporary or permanent extinction of local species [122]. However, these aspects have a lower potential for biomimetic material systems and few parallels can be drawn with other artificial material systems; they are therefore not discussed further in this paper.

### 3.2. Resilience through Self-Repair of Damage

The self-repair of wounds is a typical life-like function that has inspired scientists to develop artificial materials and material systems. Regardless of whether they have or do not have a biological model, the artificial self-repairing material systems are capable of self-repairing cracks, fractures and scratches. Rapid self-sealing immediately after the damage and the subsequent self-healing contribute substantially to the long-term structural integrity and functionality of plants, animals and human-made components, enabling them to attain their expected lifetime or lifespan. In 1970, Malinskii et al. [123] published, for the first time, studies on the self-healing of cracks. However, little progress was made until 2001 when the article of White et al. [124] was published in *Nature*. A great deal of attention was then paid to the topic of “self-healing of artificial materials” and a wave of research projects and their respective publications followed. In the context of quantifying self-repair, several questions arise that should be answered with respect to the intended application (Table 4).

**Figure 11:**
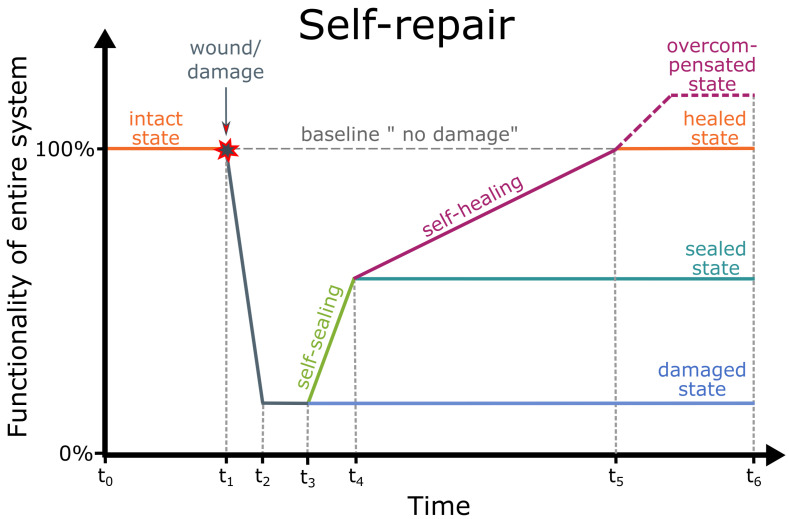
Schematic representation of biological and artificial material systems with and without the self-repair function after damage. The material system starts with an intact and undamaged state of 100% functionality (= baseline “no damage”) at time $t_{0}$. After a wounding or damaging event at time $t_{1}$ (red star), the material system continuously loses its functionality until it is in a damaged state at time $t_{2}$. Starting at time $t_{3}$, four scenarios are possible: (1) the material system has no self-repair function and remains in the damaged state, (2) the material system regains part of its functionality by self-sealing and remains in a sealed state (time $t_{4}$), (3) the material system returns to the baseline through self-healing and exhibits the initial state with 100% functionality (time $t_{5}$) and (4) the damage is sometimes overcompensated with the final state being above 100% functionality (time $t_{6}$) (e.g., lignification, scar or callus formation).

Several equations can be found in the literature that quantitatively analyse the mechanical properties of various states of the self-healing process by comparing the damaged state (freshly injured), the sealed state and the healed state with the intact state (without damage) [34,35,36,37]. These equations can be applied to wound healing in plants and animals and to the self-repair process in human-made material systems. Since the repair efficiency $\eta_{state}$ of individual material properties and thus the corresponding mechanical integrity can differ considerably, it is essential that the values of the same material property are compared in various states (intact, damaged, sealed, healed). Equation (4) gives the percentage loss of mechanical integrity through damage compared with the intact undamaged state (cf. Figure 11 at time $t_{2}$). Equations (5)–(7) calculate the percentage restoration after immediate sealing (cf. Figure 11 at time $t_{4}$), by healing only and after sealing and completed healing (cf. Figure 11 at time $t_{5}$), respectively.

(4) $$\eta_{damaged}\left(\%\right)=\frac{value_{damaged}}{value_{intact}}\cdot 100\%$$

(5) $$\eta_{sealed}\left(\%\right)=\frac{value_{sealed}}{value_{intact}}\cdot 100\%$$

(6) $$\eta_{healed}\left(\%\right)=\frac{value_{healed}-value_{sealed}}{value_{intact}}\cdot 100\%$$

(7) $$\eta_{repair}\left(\%\right)=\frac{value_{healed}}{value_{intact}}\cdot 100\%$$

Restoration of structural integrity, on the other hand, can only be assessed qualitatively by imaging techniques (e.g., light microscopy, scanning electron microscope, X-ray computed tomography). Images can be used to assess the extent to which the damage has been sealed or healed and whether the same material was used in the damaged area. Dependent on the body plan [43] of the plant species and the hierarchical level, self-sealing can be achieved by the leakage of plant sap such as mucilage (e.g., cacti [37,44]) or latex (e.g., latex-bearing plants [44,45]) into the incision gap, the expansion of turgescent parenchyma cells into (micro-)fissures (e.g., *Aristolochia* species [125]), the deformation of dermal tissues (all plants studied to date) and the deformation of the entire leaf (e.g., *Delosperma* species [46,47,48,49]). In addition to the physico-chemical self-sealing mechanisms, more complex and metabolic self-healing mechanisms have been found in plants, such as the coagulation of latex (e.g., latex-bearing plants [45]), the lignification of tissues in the wound area (e.g., flax cultivars [126]) and, in almost all plants studied to date, the formation of a (ligno-suberised) boundary layer and the development of a wound periderm [44,127].

Various bio-inspired and biomimetic self-sealing material systems and self-healing material systems have been developed in recent years [128]. Many artificial material systems have been inspired by the self-repair mechanisms found in blood [35,129,130,131,132,133]. After the skin is injured, a scar often appears after complete self-healing, which represents the overcompensated state because many mechanical properties of the scar exceed those of the intact skin. Another prime example is the development of human-made material systems inspired by the self-repair mechanisms of bones, as bone is the only living material system that does not form scars after damage [134]. Moreover, self-repairing principles have inspired scientists to develop plant-inspired material systems mostly with a self-sealing function and a few with a self-healing function [128]. However, in contrast to the biological models, the structures (e.g., tubes, capsules) for self-repairing functions must additionally be introduced into the artificial material systems. On the one hand, these additional structures can reduce the initial functionality of the material system. On the other hand, the self-repair of geometric and mechanical properties and thus the regain of functionality contributes to an extension of material and product lifetime because premature catastrophic failure of the entire system can be avoided [135].

## 4. Conclusions

The aim of this study has been to understand longevity via the concepts of robustness and resilience of functionality of material systems as expressed in plants and to learn about the potential of these concepts in the extension of material and product lifetime. Both concepts are suitable for extending the lifespan of plants and the lifetime of human-made products. This is in good agreement with the Sustainable Development Goal 12 of the 2030 Agenda and, in particular, with target 12.1 “By 2030, achieve the sustainable management and efficient use of natural resources” and target 12.5 “By 2030, substantially reduce waste generation through prevention, reduction, recycling and reuse”. Bio-inspired resource preservation and waste avoidance by extending the lifetime of human-made products is an essential contribution to sustainable development.

Although we introduce the concepts of robustness and resilience separately in the article, they can be simultaneously present in biological and artificial material systems, thus providing optimised functional reliability. At the interface between biology and technology, we can understand robustness by means of fault tolerance by remaining unharmed and resilience in the sense of tolerance towards hardware failures, software errors and human mistakes by returning to the original state of functionality. We discuss damage resistance through safety factors comparable with a series circuit of cumulating elements allowing material systems to withstand additional loads. Damage resistance of smooth transitions results from multiple superimposed geometric and mechanical gradients. Moreover, material systems can react to changing environmental conditions through response, acclimation, adaptation, adaptivity and optimisation. Here, it has become apparent that particularly the term “adaptive” has completely different meanings depending on the scientific discipline involved. Furthermore, the limits of biomimetics have been clarified, e.g., a grass stem oscillating in the wind is not suitable as a model for a tower with meter-wide oscillation amplitudes, if people are to live, work or eat in it. Interestingly, “training”, for example by cyclic loading, can take place in biological and artificial material systems resulting in “trained” plants and products.

Moreover, we define resilience in the sense of tolerance towards failures of system parts or damage to the material system by its return to its original state. At the biology–technology interface, we introduce various types of redundancy. As in a parallel circuit, if one element fails, its function can be taken over by other elements of a system with little or no loss of overall functionality. In the case of a wound or some form of damage, rapid self-sealing and subsequent long-term self-healing can recover structural and mechanical integrity and, thus, the functionality of the material systems.

The functionalities described, including the examples presented from biological and biomimetic material systems, together with the definitions from the glossary, provide a comprehensive toolbox for comparative analyses and interdisciplinary collaborations between biologists and engineers enabling them to develop and advance material systems with a great longevity potential.

Our fundamental concept can serve as an important tool for future topics because both robustness and resilience can be represented as functionality as a function of time and can thus be both compared and combined with each other. The use of dimensionless quantities is of great advantage, since further characteristics (e.g., geometric, mechanical, structural properties and trade-offs such as the twist-to-bend ratio), additional states, various types of perturbations (e.g., wind storms, earthquake, ageing, pandemic) and further principles (e.g., compartmentalisation, complexity, reliability, modularity) in various fields (e.g., health, economy, civil engineering, manufacturing) can be easily quantified with our fundamental concept.

## 5. Glossary

Being aware of the different understandings and interpretations of the various terms from the broad topic of longevity, both between and within different research disciplines, the aim of this glossary is not to claim a universal validity of the definitions. Instead, we provide a guideline and orientation for a common understanding and for simplified and targeted collaborations in the field of biomimetic material systems.

- **Acclimation/acclimatisation:** Acclimation of individual plants to environmental constraints takes place over a time period of days and weeks, resulting in changes of gene expression and thus altered morphological, anatomical and mechanical properties [89], leading to “trained” plants. In the materials science context, this is usually referred to as adaptivity.
- **Adaptation:** In a biological context, adaptation is the result of genetic change in populations over evolutionary time [80]. In engineering, optimisation of systems can be achieved via evolutionary algorithms or machine learning within several generations or epochs.
- **Adaptive system:** “An adaptive system is a set of elements which interact with each other and has at least one process which controls the system’s adaptation, that is, the correlation between structure, function or behaviour and its environment, to increase its efficiency to achieve its goals”. ([136], p. 760)
- **Bio-inspiration:** Transfer of an idea derived from living organisms into a technical application [128].
- **Biomimetics:** Transfer of a functional principle derived from living organisms into a technical application [2,128].
- **Failure:** Loss of load carrying capacity in materials or structural elements (with brittle, ductile or intermediate behaviour) [137].
- **Fault:** A material system or structural element that can no longer satisfy the user requirements [138]. In technology, faults mean a break or a defect of the hardware; in computer science, errors occur in the software or calculations, whereas a mistake is caused by a human being.
- **Function:** In a biological context, being understood in the sense of traits evolved to contribute to fitness [139]. In a technical context, being understood in the sense of a specific process, action or task [140].
- **Functionality:** The quality or status of being functional [141].
- **Functional principle:** The underlying principle for executing a function. Sometimes also referred to as operating principle.
- **Lifespan:** The period between germination and death of a plant.
- **Lifetime:** The period between the production of a product and its being discarded [14].
- **Longevity:** The attainment of the species-specific lifespan of biological organisms by ensuring the survival of the plant species through the release of spores or seeds. In an engineering context, the attainment of the consumer-expected lifespan of material systems without loss of functionality.
- **Material system:** Through the structural and functional interaction of various materials, a material system emerges with different chemical and physical properties, new functions and other functionalities compared with the individual materials.
- **Obsolescence:** The ageing of a product or artificial material system, causing it to lose functionality and usability. An extreme case is planned obsolescence, in which a shortening of the lifetime of a product is accepted or actively implemented by the manufacturer [20].
- **Property:** Properties or characteristics of materials, technical components or plant organs are measured via standardised or established test methods. Mechanical properties characterise the reaction to applied loads; geometric properties include cross-sectional and volumetric geometry, size and shape.
- **Resilience:** Ability of a system to manage damage by returning to its original functionality after (partial) failures have occurred (modified after [142]).
- **Resistance:** The capability of a system to withstand harmful events without significant damage and to remain unharmed or unaffected (modified after [143]).
- **Redundancy:** A system in which the functional failure of one (or more) contributing elements can be compensated by other elements and thus the functionality of the overall system can be maintained.
  - **Cold redundancy:** Redundant system in which one (or more) elements are passive and take over the function of one (or more) elements in case of their failure [112]. Also known as standby redundancy.
  - **Hot redundancy:** Redundant system, with all involved elements being active at the same time and taking over the failure of one (or more) elements [108]. Also known as active redundancy.
- **Response:** Immediate response of plants, such as wind-induced reconfiguration of leaves, branches or entire plants occurs within seconds to minutes [88].
- **Robustness:** The capability of a system to prevent damage and to maintain its functionality even if unforeseen faults (e.g., in hardware) or errors (e.g., in software) occur or a mistake is made by a human being [144].
- **Safety factor:** A dimensionless factor or percentage that describes the multiple by which the structure can carry more than their actual static load.
- **Self-repair:** Generic term that encompasses self-sealing and self-healing in biological and technical systems [41,145].
  - **Self-healing:** Longer-lasting self-healing phase (after self-sealing) in which the fissures are structurally repaired and thus are no longer present and the mechanical properties in the damaged area are (at least partially) restored [41,145].
  - **Self-sealing:** Self-sealing takes place immediately after damage, resulting in a fissure that remains but that is functionally repaired; the mechanical properties in the damaged area have, however, not yet been restored [41,145].
- **Senescence:** Senescence is a degeneration process during the ageing of plants, marking the last phase of a developmental program. It occurs in a signal-controlled and time-coordinated manner [146].
- **Structural materials:** Structural materials are structured in complex hierarchical architectures at various scales, making it possible to combine lightweight construction with good strength and toughness properties. A wide variety of these material systems can be found in nature (e.g., bamboo or bones); however, implementation in synthetic structures is currently only possible at high expense [27].

## Figures and Tables

**Table 1:** Damage prevention through reactions of plants (according to [80]).

BOTANY	Response	Acclimation	Adaptation
Subject	Individual plants	Individual plants	Populations of plants
Effect	Reconfiguration of plant organs	Change of gene expression	Change of genetic information
Time span	Seconds to minutes	Days to months	Evolutionary time
Result	“Re-oriented” plants	“Trained” plants	“Adapted” plants
Plant example	Reversible streamlining of plants and plant organs under wind loads [82,83,84]	Non-hereditary alteration of growth pattern (e.g., reduction in shoot elongation) [81,85,86,87,88,89]	Variation of hereditary traits (e.g., trampling tolerant plants with low growth and rosette formation) [90]

**Table 2:** Damage prevention through reactions of human-made components and designs.

TECHNOLOGY	Response	Adaptivity	Optimisation
Subject	Individual components	Individual components	Populations of designs
Effect	Reconfiguration of component parts	Change of material properties	Optimisation of design parameters
Time span	Seconds to days	Number of cycles	Number of generations
Result	“Re-oriented” components	“Trained” components	“Optimised” design
Biomimetic example	Stimulus-responsive buildings (e.g., Urbach Tower) and facade-shading systems (e.g., Flectofin, Flectofold, Hygroskin) [91,92,93,94,95,96]	Persistent change of material properties through cyclic loading [97,98]	Optimised parameters through evolutionary algorithms (e.g., coffee blend, winglets for air planes, lightweight constructions) [99,100,101,102,103,104]

**Table 3:** Comparison of adaptation of biological evolution and evolutionary strategy used in technology (modified after [104]).

	Evolution (Biology)	Evolutionary Strategy (Technology)
Subject	Living being	Object to be optimised
Mutation	Random change of genetic information	Random change of input variables (= object parameters)
Recombination	Reshuffling of parental genetic material (e.g., meiosis)	New combination of parental object parameters
Selection	Selection of those individuals with the best fit to the natural environment	Selection of those individuals that best meet the optimisation criterion
Result	Adapted organism	Optimised object

**Table 4:** Questions that arise in the context of restoring the mechanical performance and structural integrity of self-repairing material systems after the occurrence of damage. Suitable answers depend on the specifications of the product.

Questions	Answers
What is being restored?	Geometric properties, mechanical properties, structural integrity, functionality of the entire system
What size of damage can be self-repaired?	Length, width and depth or radius (e.g., millimetres, centimetres)
How quickly should self-repair be carried out?	Seconds, days, weeks, month, years
How often can the damage self-repair?	Once, twice, several times
Should self-repair be initiated autonomously or by a specific trigger?	Autonomous self-repair without a trigger or non-autonomous self-repair initiated by a specific trigger (e.g., temperature, light, humidity, mechanical compression)
How can the structural integrity of the damaged and repaired status be measured?	Qualitative assessment by imaging techniques (e.g., light microscopy, SEM, X-ray tomography)
How can the mechanical integrity of the damaged and repaired status be measured?	Quantitative analyses by various equations [34,35,36,37]

## Data Availability

Not applicable.
